# Measuring Vaccine Efficacy Against Infection and Disease in Clinical Trials: Sources and Magnitude of Bias in Coronavirus Disease 2019 (COVID-19) Vaccine Efficacy Estimates

**DOI:** 10.1093/cid/ciab914

**Published:** 2021-10-26

**Authors:** Lucy R Williams, Neil M Ferguson, Christl A Donnelly, Nicholas C Grassly

**Affiliations:** MRC Centre for Global Infectious Disease Analysis, School of Public Health, Imperial College London, London, United Kingdom; MRC Centre for Global Infectious Disease Analysis, School of Public Health, Imperial College London, London, United Kingdom; MRC Centre for Global Infectious Disease Analysis, School of Public Health, Imperial College London, London, United Kingdom; Department of Statistics, University of Oxford, Oxfordshire, United Kingdom; MRC Centre for Global Infectious Disease Analysis, School of Public Health, Imperial College London, London, United Kingdom

**Keywords:** COVID-19, SARS-CoV-2, vaccine efficacy, bias, Clinical Trial

## Abstract

**Background:**

Phase III trials have estimated coronavirus disease 2019 (COVID-19) vaccine efficacy (VE) against symptomatic and asymptomatic infection. We explore the direction and magnitude of potential biases in these estimates and their implications for vaccine protection against infection and against disease in breakthrough infections.

**Methods:**

We developed a mathematical model that accounts for natural and vaccine-induced immunity, changes in serostatus, and imperfect sensitivity and specificity of tests for infection and antibodies. We estimated expected biases in VE against symptomatic, asymptomatic, and any severe acute respiratory syndrome coronavirus 2 (SARS-CoV-2) infections and against disease following infection for a range of vaccine characteristics and measurement approaches, and the likely overall biases for published trial results that included asymptomatic infections.

**Results:**

VE against asymptomatic infection measured by polymerase chain reaction (PCR) or serology is expected to be low or negative for vaccines that prevent disease but not infection. VE against any infection is overestimated when asymptomatic infections are less likely to be detected than symptomatic infections and the vaccine protects against symptom development. A competing bias toward underestimation arises for estimates based on tests with imperfect specificity, especially when testing is performed frequently. Our model indicates considerable uncertainty in Oxford-AstraZeneca ChAdOx1 and Janssen Ad26.COV2.S VE against any infection, with slightly higher than published, bias-adjusted values of 59.0% (95% uncertainty interval [UI] 38.4–77.1) and 70.9% (95% UI 49.8–80.7), respectively.

**Conclusions:**

Multiple biases are likely to influence COVID-19 VE estimates, potentially explaining the observed difference between ChAdOx1 and Ad26.COV2.S vaccines. These biases should be considered when interpreting both efficacy and effectiveness study results.

## Teaser

Multiple biases have the potential to influence vaccine efficacy estimates, with their direction and magnitude dependent on the vaccine properties and testing strategies. We expect vaccine efficacy against infection to be slightly underestimated in the coronavirus disease 2019 vaccine trials.

The coronavirus disease (COVID-19) phase III vaccine trials have demonstrated efficacy against symptomatic infection for multiple vaccines, with estimates ranging from 50% to 95% [1]. Yet a vaccine that protects against symptomatic disease may work by preventing infection (infection-blocking vaccine), by preventing progression to symptoms upon infection (disease-blocking vaccine), or by a combination of these 2 mechanisms (Supplementary Figure 1) [2]. Understanding the extent to which the COVID-19 vaccines protect against infection is important because the success of their vaccination programs is highly contingent not only on symptomatic cases but also asymptomatic infection and community transmission [3].

The predominant primary outcome of the COVID-19 vaccine trials is vaccine efficacy (VE) against the first case of polymerase chain reaction (PCR)-confirmed symptomatic disease, *VE*_*sym*_. This is measured by PCR-testing trial participants with COVID-19 symptoms and is sensitive to the clinical case definition [4]. As secondary outcomes, most trials also measure the incidence of asymptomatic infections, using either (i) regular swabbing and PCR testing, or (ii) serological testing for anti-nucleocapsid antibodies at prespecified time intervals, which allows seroconversion after infection to be identified for vaccines based on the spike protein (Table 1). Both strategies allow for estimation of VE against asymptomatic infection (*VE*_*asym*_) and VE against any infection (*VE*_*in*_).

**Table 1.** Methods for Measuring Vaccine Efficacy Against Infection, Symptomatic Infection and Asymptomatic Infection in the COVID-19 Phase III Trials

Vaccine Efficacy Outcome	Measurement Method	Vaccine^b^	
		Spike Protein	Whole Virus^c^
Symptomatic infection	PCR swabbing upon self-reporting of symptoms	Oxford-AstraZeneca ChAdOx1 Janssen Ad26.COV2.S GRI Sputnik V Pfizer-BioNTechBNT162b2 Moderna mRNA-1273 Novavax NVX-CoV2373	Bharat Biotech Covaxin Sinopharm BBIBP-CorV Sinovac CoronaVac
Asymptomatic infection	Regular PCR swabbing regardless of symptoms	Oxford-AstraZeneca ChAdOx1^a^	Bharat Biotech Covaxin
	Serology testing at prespecified time intervals	Oxford-AstraZeneca ChAdOx1 Janssen Ad26.COV2.S Pfizer-BioNTechBNT162b2 Moderna mRNA-1273 Novavax NVX-CoV2373	
Overall infection	Serologically confirmed asymptomatic infections + PCR-confirmed symptomatic infections	Janssen Ad26.COV2.S Moderna mRNA-1273 Novavax NVX-CoV2373	Sinovac CoronaVac
	Serologically confirmed asymptomatic infections + serologically confirmed symptomatic infections	Oxford-AstraZeneca ChAdOx1 Janssen Ad26.COV2.S Moderna mRNA-1273 GRI Sputnik V Novavax NVX-CoV2373	Sinovac CoronaVac
	PCR-confirmed asymptomatic infections + PCR-confirmed symptomatic infections	Oxford-AstraZenecaChAdOx1	Bharat Biotech Covaxin

Abbreviations: COVID-19, coronavirus disease 2019; GRI, Gamaleya Research Institute; mRNA, messenger RNA; PCR, polymerase chain reaction; SARS-CoV-2, severe acute respiratory syndrome coronavirus 2.

COV002 (UK) trial only.

Vaccines only listed where measurement approach has been reported. Trial numbers provided in Supplementary Table 1.

For vaccines based on the whole virus, it may be possible to infer infection via a rise in SARS-CoV-2 antibodies following a period after vaccination.

*VE*_*asym*_ is a complex outcome due to its relationship with the 2 mechanisms of VE. A vaccine that protects only against infection will reduce the number of symptomatic and asymptomatic infections in equal proportions, leading to a positive *VE*_*asym*_. Yet a vaccine that protects against symptom development will convert symptomatic cases to asymptomatic, potentially giving a negative *VE*_*asym*_. The counterintuitive interpretation of this outcome has been noted [2, 5], but the relationship between *VE*_*asym*_, *VE*_*in*_, and VE against progression to symptoms (*VE*_*pr*_) has not been quantified.

Estimates of VE are known to be biased by factors such as imperfect test sensitivity and specificity and the accumulation of immunity over time [6–8]. However, there has been little discussion on the potential biases of the COVID-19 VE estimates [9, 10]. We developed a mathematical model of a vaccine trial to investigate the factors affecting observed values of VE. We first illustrate parameters affecting measured *VE*_*asym*_, then quantify the influence of different biases on VE estimates, notably the impact of (i) the build-up of immunity from undetected asymptomatic infections, (ii) imperfect test sensitivity and specificity for alternative testing strategies, (iii) differential detection of asymptomatic and symptomatic infections, and (iv) confounding of VE and probability of symptoms by age. We finish by estimating bias-adjusted VEs for 2 COVID-19 vaccines.

## METHODS

### Analytical Derivations

VE is defined as 1-RR, where RR is some measure of the relative risk in the vaccine compared with the control arm [11]. For most primary and secondary outcomes of the COVID-19 vaccine trials, the relative risk is based on an incidence rate ratio (IRR) such that


$$\begin{array}{l} VE=1-IRR=1-\frac{\begin{array}{c} number\;outcomes\\ (vaccine)\;/\;person\text{-}years\\ observation\;(vaccine) \end{array}}{\begin{array}{c} number\;outcomes\\ (control)/\;person\text{-}years\\ observation\;(control) \end{array}}=1-\frac{IR_{v}}{IR_{c}} \end{array}$$

where *IR*_*v*_ and *IR*_*c*_ are the incidence rate in the vaccine and control groups respectively.

For outcomes measured at fixed time points (eg, seroconversions), the relative risk can be calculated using the cumulative incidence ratio (CIR) such that


$$\begin{array}{l} VE=1-CIR=1-\frac{\begin{array}{c} cumulative\;number\\ seroconverted\;(vaccine)\\ /\;number\;tested\;(vaccine) \end{array}}{\begin{array}{c} cumulative\;number\\ seroconverted\;(control)\\ /\;number\;tested\;(control) \end{array}}=1-\frac{CI_{v}}{CI_{c}} \end{array}$$

where *CI*_*v*_ and *CI*_*c*_ are the cumulative incidence in the vaccine and control groups respectively. For a “leaky” vaccine, VE based on cumulative incidence approximates that based on the incidence rate for low incidence or short follow-up periods but biases toward zero as follow-up time and incidence increase [8].

For vaccines that protect against infection and/or symptoms, VE against symptomatic infection is given by


$$\begin{array}{l} VE_{sym}=1-(1-VE_{in})(1-VE_{pr}) \end{array}$$ (1)

[12]. VE against asymptomatic infection depends on the incidence of asymptomatic infections that are not prevented by the vaccine and on symptomatic infections that the vaccine prevents from progressing, such that


$$\begin{array}{l} VE_{asym}=1-\frac{(1-p_{s})\left(1-VE_{in}\right)+p_{s}\left(1-VE_{in}\right)VE_{pr}}{\left(1-p_{s}\right)} \end{array}$$ (2)

where *p*_*s*_ is the probability of symptoms in the absence of vaccination. Substituting equation 1 into equation 2 allows *VE*_*in*_ to be derived as a simple function of *VE*_*sym*_ and *VE*_*asym*_


$$\begin{array}{l} VE_{in}=\;(1-p_{s})VE_{asym}+p_{s}VE_{sym} \end{array}$$ (3)

Rearranging equation 1 and substituting equation 3 into equation 1 then provides a solution for *VE*_*pr*_


$$\begin{array}{l} VE_{pr}=\;1-\frac{1-VE_{sym}}{1-((1-p_{s})VE_{asym}+p_{s}VE_{sym})} \end{array}$$ (4)

If asymptomatic infections are less likely to be detected than symptomatic infections, and a vaccine is protective against symptom development (*VE*_*pr*_ > 0), then observed *VE*_*in*_ ≠ true *VE*_*in*_. The observed *VE*_*in*_ depends on the relative incidence of detected infections, and can be related to the true efficacy by


$$\begin{array}{l} Observed\;VE_{in}=\;VE_{in}+\frac{p_{s}\left(1-\sigma \right)\left(1-VE_{in}\right)VE_{pr}}{1-(1-p_{s})(1-\sigma )} \end{array}$$ (5)

where σ represents the relative probability of asymptomatic to symptomatic infection detection. For intermediate steps for equations 3–5 and estimation of confidence intervals for *VE*_*in*_ and *VE*_*pr*_; see Supplementary Methods.

Analytical solutions become more complex when incorporating additional biases, so we developed a stochastic mathematical (cohort) model of a phase III vaccine trial.

### Mathematical Model

The model follows a susceptible, infected, recovered (SIR) structure, implemented as a Markov model, and allows for asymptomatic and symptomatic infections, natural immunity, changes in serostatus and imperfect test sensitivity and specificity. We assume a constant infection rate over time and a “leaky vaccine” model, so each vaccinated individual’s probability of infection is reduced by *VE*_*in*_ and their risk of then developing symptoms is reduced by *VE*_*pr*_. We assume no heterogeneity in population characteristics but perform a sensitivity analysis to assess the effect of variation in *p*_*s*_ and VE by age.

We model 2 testing approaches for asymptomatic infections: (i) weekly PCR testing and (ii) serological testing at 1, 2, 6, 12, and 24 months after baseline. We assume that responsive PCR testing detects all symptomatic infections. Observed VE is calculated from the simulated incidence of detected infections in each trial arm. Efficacy is estimated as 1-IRR for all outcomes except those estimated using serology, for which efficacy is estimated as 1-CIR, using the cumulative number of seroconversions detected in each serology assessment up to the present time interval. Point estimates and confidence intervals are given by the mean and 2.5 and 97.5 percentiles of 1000 simulated estimates.

### Application to COVID-19

Applying the model to COVID-19, we assumed a natural probability of developing symptoms upon infection of 0.67 [13], a serology test specificity of 99.84% [14], and sensitivity of 95% and 80% to symptomatic and asymptomatic infections, respectively [15]. We used data on the probability of PCR detection over time since infection for individuals without symptoms [16] to estimate the probability of detecting an asymptomatic infection with weekly PCR swabbing (Supplementary Table 2) and assumed a PCR test specificity of 99.945% [17]. We used the model to estimate bias-adjusted VE estimates for 2 adenovirus vector vaccines with published trial data on asymptomatic infection, ChAdOx1 (Oxford-AstraZeneca) and Ad26.COV2.S (Janssen). We used our best parameter estimates to estimate the infection rate from the number of reported infections in the placebo arm, accounting for imperfect test characteristics. We ran the model under a range of true *VE*_*in*_ and *VE*_*pr*_ values to find which combination gave the trial-reported estimates, then generated 95% uncertainty intervals (UI) using Latin hypercube sampling to give the range within which the VE is expected to lie, considering both statistical variation and parameter uncertainty. We then used rank regression to evaluate the contribution of individual parameters to the biases (Supplementary Methods).

The model is described further in Supplementary Methods and Supplementary Figures 2 and 3. Model parameters are provided in Supplementary Table 3. Code is available at: https://github.com/lucyrose96/COVID-19-Trial-Model.

## RESULTS

### Interpretation of Vaccine Efficacy Against Asymptomatic Infection

**Figure 1.**
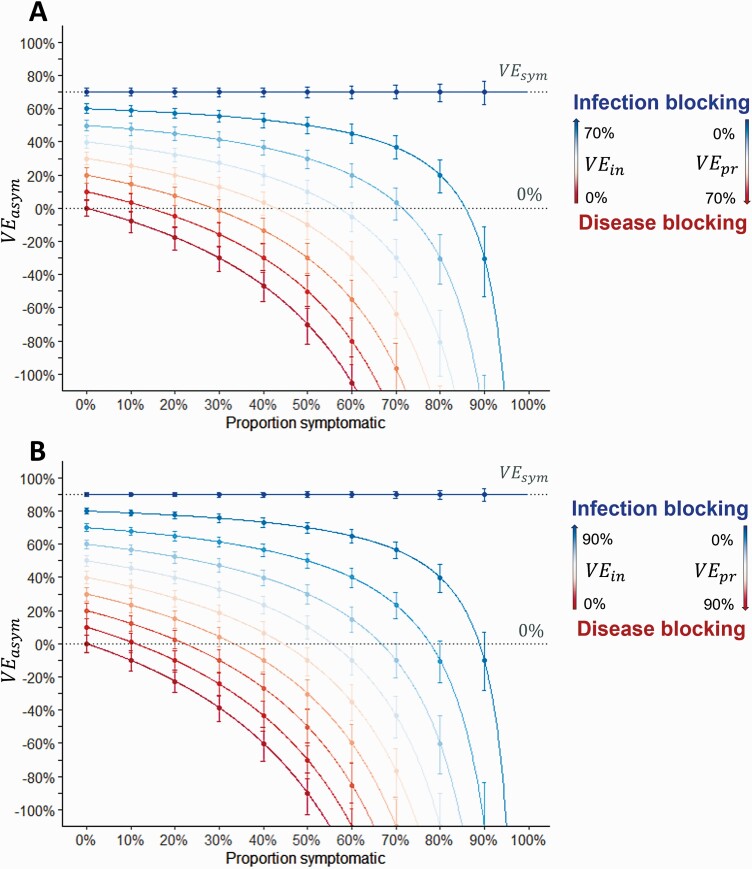
Estimated vaccine efficacy against asymptomatic infection. Red to blue gradient represents the transition from a disease-blocking vaccine (*VE*_*in*_ = 0% and *VE*_*pr*_ = *VE*_*sym*_) to an infection-blocking vaccine (*VE*_*in*_ = *VE*_*sym*_ and *VE*_*pr*_ = 0%). Each successive line represents a 10% increase in *VE*_*in*_ and a corresponding decrease in *VE*_*pr*_ (such that *VE*_*sym*_ remains the same). Lines show predicted values from equation 2; points and error bars show the observed mean and 2.5 and 97.5 percentiles from 1000 simulations, with efficacy calculated as 1-IRR, censoring after the first infection. *A*, Vaccine with 70% efficacy against symptomatic infection (eg, Oxford-AstraZeneca/Janssen vaccines). *B*, Vaccine with 90% efficacy against symptomatic infection (eg, Pfizer/Moderna vaccines). Abbreviations: IRR, incidence rate ratio; *VE*_*asym*_, vaccine efficacy against asymptomatic infection; *VE*_*in*_, vaccine efficacy against infection; *VE*_*pr*_, vaccine efficacy against progression to symptoms; *VE*_*sym*_, vaccine efficacy against symptomatic infection.

Observed *VE*_*asym*_ was positively associated with *VE*_*in*_ but negatively associated with *VE*_*pr*_ and the proportion of infections that were symptomatic (Figure 1). For vaccines that only prevented infection, *VE*_*asym*_ was equal to *VE*_*sym*_. For vaccines with efficacy predominantly mediated by prevention of symptoms, *VE*_*asym*_ was low or negative, particularly when a large proportion of infections were naturally symptomatic. For vaccines with high *VE*_*sym*_ (Figure 1A), protection against infection can be expected with lower values of *VE*_*asym*_ than vaccines with moderate *VE*_*sym*_ (Figure 1B). *VE*_*in*_ and *VE*_*pr*_ could be estimated from *VE*_*sym*_ and *VE*_*asym*_ using equations 3 and 4 (Supplementary Figure 4).

### Possible Biases in COVID-19 Vaccine Trials

**Figure 2.**
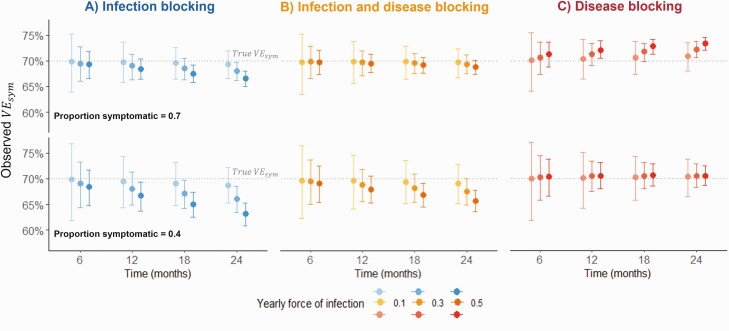
Change in estimated vaccine efficacy against symptomatic infection over a 2-year follow-up. Points and error bars show the observed mean and 2.5 and 97.5 percentiles from 1000 simulations, with efficacy calculated as 1-IRR, censoring after the first symptomatic infection. Time = 0 months represents 2 weeks post second dose. Sensitivity and specificity = 100%. True vaccine efficacies: *VE*_*sym*_ = 70%. *A*, *VE*_*in*_ = 70%; *VE*_*pr*_ = 0%. *B*, *VE*_*in*_ = 50%, *VE*_*pr*_ = 40%. *C*, *VE*_*in*_ = 0%, *VE*_*pr*_ = 70%. Abbreviations: IRR, incidence rate ratio; ; *VE*_*asym*_, vaccine efficacy against asymptomatic infection; *VE*_*in*_, vaccine efficacy against infection; *VE*_*pr*_, vaccine efficacy against progression to symptoms; *VE*_*sym*_, vaccine efficacy against symptomatic infection.

The build-up of immunity from undetected asymptomatic infections caused *VE*_*sym*_ to bias in opposite directions for infection-blocking and disease-blocking vaccines. For infection-blocking vaccines, estimated *VE*_*sym*_ decreased over time, with greater decreases observed for higher infection rates and lower probabilities of symptoms (Figure 2A). For disease-blocking vaccines, a downward bias was only observed when the probability of symptoms was low (Figure 2C). Instead, for most combinations of parameters, estimated *VE*_*sym*_ increased slightly over time. For an infection and disease-blocking vaccine (50% *VE*_*in*_, 40% *VE*_*pr*_), a small downward bias was observed (Figure 2B). The biases were sensitive to the VE calculation, as *VE*_*sym*_ estimated with cumulative incidence decreased over time for all vaccine profiles (Supplementary Figure 5).

**Figure 3.**
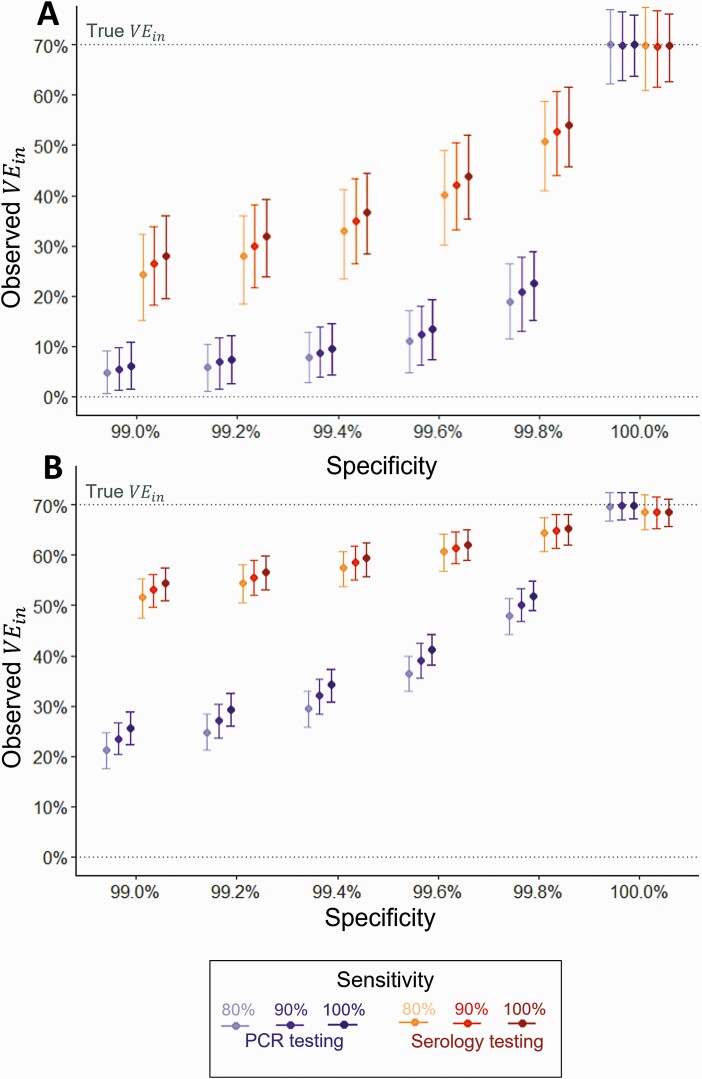
Impact of imperfect test sensitivity and specificity on serology-estimated and PCR-estimated vaccine efficacy against infection. *A*, Low force of infection (5% per year). *B*, High force of infection (30% per year). At 6-month follow-up visit: serology tests taken at month 1, 2, and 6 (cumulative seroconversions up to 6-month visit); PCR tests taken weekly. Serology efficacy calculated using 1-CIR; PCR efficacy calculated using 1-IRR. Sensitivity assumed to be equal for symptomatic and asymptomatic infections. Points and error bars represent the mean and 2.5 and 97.5 percentiles from 1000 simulations. At 100% specificity, a slight bias is observed for the serology-estimated *VE*_*in*_ because estimates based on the CIR bias toward zero over time, particularly in high incidence settings [8]. Abbreviations: CIR, cumulative incidence ratio; IRR, incidence rate ratio; PCR, polymerase chain reaction; *VE*_*in*_, vaccine efficacy against infection.

Imperfect test characteristics biased efficacy estimates toward zero. Factors increasing the magnitude of the bias were: reduced specificity, reduced sensitivity, increased testing frequency, and calculation with the CIR instead of the IRR. Although the serology estimated *VE*_*in*_ was based on the CIR (as person-time at risk is unknown), the bias was usually lower than the weekly-PCR estimate, for a given sensitivity and specificity, due to the lower frequency of testing (Figure 3). This led to substantial bias particularly in low incidence settings. For example, with a high specificity (99.8%) and sensitivity (100%), a true *VE*_*in*_ of 70% in a low incidence setting (5% per year) was underestimated at 23%.

**Figure 4.**
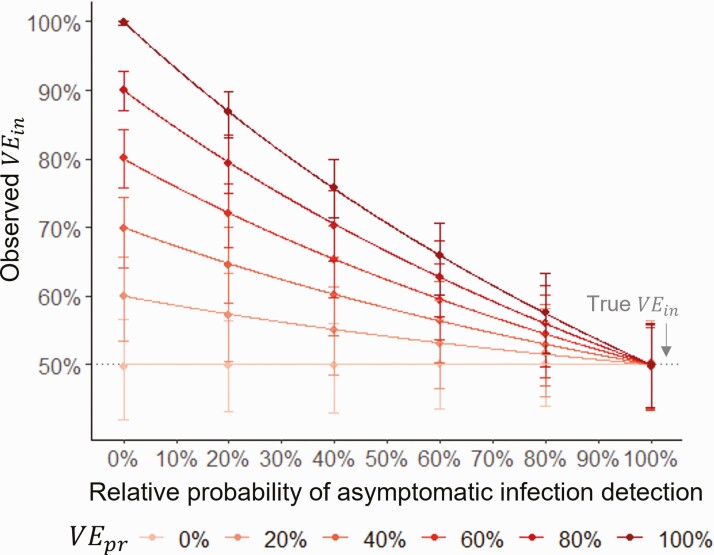
Impact of differential detection of asymptomatic and symptomatic infections on observed vaccine efficacy against infection. Lines show values estimated with equation 4, points and error bars show the observed mean and 2.5 and 97.5 percentiles from 1000 simulations, with efficacy calculated as 1-IRR, censoring after the first infection. Specificity = 100%, yearly force of infection = 5%, follow-up = 12 months from 2 weeks post 2nd dose. Abbreviations: IRR, incidence rate ratio; *VE*_*in*_, vaccine efficacy against infection; *VE*_*pr*_, vaccine efficacy against progression to symptoms.

For a vaccine that was protective against symptom development (*VE*_*pr*_ > 0), *VE*_*in*_ was overestimated when asymptomatic infections were less likely to be detected than symptomatic infections (Figure 4). The greater the difference in the detection probabilities and the greater the vaccine’s protection against symptoms, the greater the overestimation.

**Figure 5.**
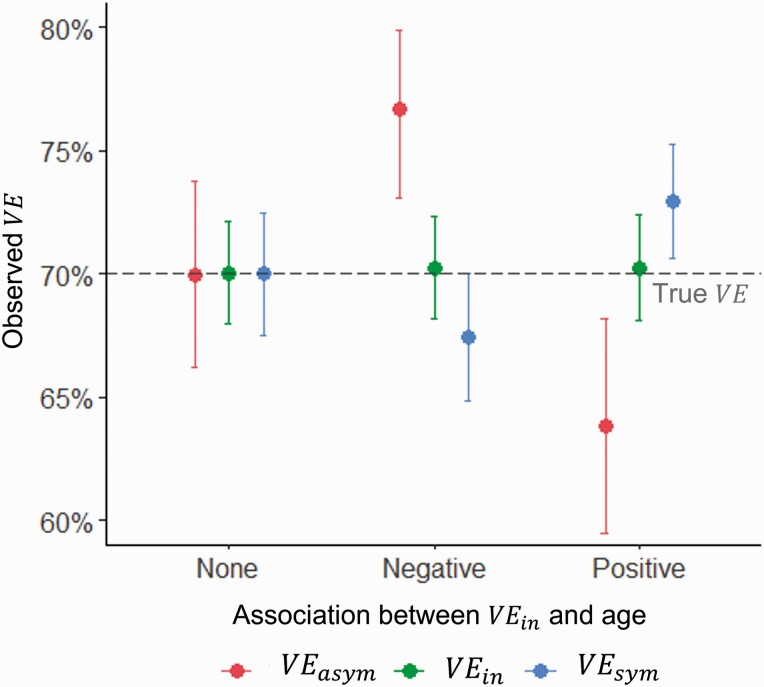
Estimated vaccine efficacy in a population with a higher probability of symptoms with age. Points and error bars represent the mean and 2.5 and 97.5 percentiles of 1000 simulations. True *VE*_*asym*_ = true *VE*_*in*_ = true *VE*_*sym*_ = 70%. Specificity = 100%, yearly force of infection = 20%, follow-up = 12 months from 2 weeks post 2nd dose. Abbreviations: *VE*_*asym*_, vaccine efficacy against asymptomatic infection; *VE*_*in*_, vaccine efficacy against infection; *VE*_*sym*_, vaccine efficacy against symptomatic infection

These results were insensitive to adding age stratification to the probability of symptoms. However, also adding age-stratification to VE led to biased *VE*_*sym*_ and *VE*_*asym*_ estimates, when not adjusted for age (Figure 5). When VE decreased with age and the probability of symptoms increased, *VE*_*asym*_ was overestimated and *VE*_*sym*_ underestimated, while the opposite was observed when efficacy increased with age. The magnitude of the difference was greater with an increased association between age and the probability of symptoms, and between age and efficacy.

### Estimating VE_in_, VE_pr,_ and the Likely Bias From the Published Trial Results

Applying equations 3 and 4 to the reported trial results gave an estimated *VE*_*pr*_ for ChAdOx1 of 43.6% (95% confidence interval [CI] 20.6–59.9) (Table 2). For Ad26.COV2.S, *VE*_*in*_ was estimated at 66.2% (95% CI 55.9–74.1), and *VE*_*pr*_ just 0.9% (95% CI −46.8 to 33.2).

**Table 2.** Estimated Vaccine Efficacy Against Infection (*VE*_*in*_) and Vaccine Efficacy Against Progression to Symptoms (*VE*_*pr*_) Using Trial-reported Vaccine Efficacy Against Symptomatic Infection (*VE*_*sym*_) and Vaccine Efficacy Against Asymptomatic Infection (*VE*_*asym*_)

Vaccine	Reported Vaccine Efficacy (%)			Calculated Vaccine Efficacy (%)		Bias Adjusted Vaccine Efficacy (%)		
	*VE*_*sym*_	*VE*_*asym*_	*VE*_*in*_	*VE*_*in*_	*VE*_*pr*_	*VE*_*asym*_	*VE*_*in*_	*VE*_*pr*_
Oxford-AstraZeneca ChAdOx1^a^ [18]	72.3	14.6	50.9	…	43.6	27.4	59.0	31.5
	63.1 to 79.3	–12.1 to 34.9	41.0 to 59.0		20.6 to 59.9	–32.1 to 87.4	38.4 to 77.1	–20.7 to 55.0
Janssen Ad26.COV2.S^b,^^c^ [19]	66.5	65.5	…	66.2	0.9	79.2	70.9	–15.2
	55.5 to 75.1	39.9 to 81.1		55.9 to 74.1	–46.8 to 33.2	14.6 to 99.0	49.8 to 80.7	–73.3 to 33.2
Pfizer-BioNTech BNT162b2^d^ [20]	95.0	…	…	…	…	…	…	…
	90.3 to 97.6							
Moderna mRNA-1273^d^ [21]	94.1	…	…	…	…	…	…	…
	89.3 to 96.8							
Novavax NVX-CoV2373^d^ [22]	96.4	…	…	…	…	…	…	…
	73.8 to 99.5							
Sinovac CoronaVac^d^ [23]	83.5	…	…	…	…	…	…	…
	65.4 to 92.1							
Sinopharm BBIBP-CorV^d^ [24]	78.1	…	…	…	…	…	…	…
	64.8 to 86.3							
Gamaleya Research Institute Sputnik V^d^ [25]	91.6	…	…	…	…	…	…	…
	85.6 to 95.2							
Bharat Biotech Covaxin^d^ [26]	80.6	…	…	…	…	…	…	…
	…							

Trials that have not yet reported an estimate are left blank. *VE*_*in*_ and *VE*_*pr*_ calculated using equations 3 and 4, respectively.

Abbreviations: mRNA, messenger RNA; PCR, polymerase chain reaction; *VE*_*asym*_, vaccine efficacy against asymptomatic infection; *VE*_*in*_, vaccine efficacy against infection; *VE*_*pr*_, vaccine efficacy against progression to symptoms; *VE*_*sym*_, vaccine efficacy against symptomatic infection..

*VE*_*sym*_ measured with responsive PCR testing of symptomatic participants, *VE*_*asym*_ measured using weekly PCR testing of asymptomatic participants, *VE*_*in*_ estimated from all positive PCR tests collected via the alternative testing strategies for symptomatic and asymptomatic infections.

*VE*_*sym*_ measured with responsive PCR testing of symptomatic participants, *VE*_*asym*_ measured using serology testing of asymptomatic participants.

*VE*_*in*_ and *VE*_*pr*_ based on odds ratio, assuming confidence intervals are normal on the log scale.

*VE*_*sym*_ measured with responsive PCR testing of symptomatic participants.

Incorporating the aforementioned biases, the model gave best estimates for ChAdOx1 *VE*_*in*_, *VE*_*asym*_ and *VE*_*pr*_ of 59.0% (95% UI 38.4–77.1), 27.4% (95% UI −32.1 to 87.4) and 31.5% (95% UI −20.7 to 55.0) respectively. While, for Ad26.COV2.S, the corresponding bias-adjusted estimates were 70.9% (95% UI 49.8–80.7), 79.2% (95% UI 14.6–99.0). and −15.2% (95% UI −73.3 to 33.2). The biases were most sensitive to the test specificity, infection rate, and testing adherence (Supplementary Tables 4–6).

## DISCUSSION

Accurately estimating COVID-19 VE outcomes is important to understand vaccine benefits, their likely impact on transmission and the long-term prospects for disease control. Simulating a COVID-19 vaccine trial helps to characterize the likely influence of biases and may help to explain differences seen between vaccines, trials, and populations.

We first derived the relationship between *VE*_*asym*_ with efficacy against infection and against disease in breakthrough infections. While increasing *VE*_*in*_ increased *VE*_*asym*_, increasing *VE*_*pr*_ decreased *VE*_*asym*_ because more infections were prevented from becoming symptomatic. This influence of *VE*_*pr*_ was stronger when the probability of symptoms was higher. Therefore, although counterintuitive, for COVID-19 where a minority of infections present asymptomatically and the vaccines have high efficacies against symptomatic infection, protection against infection can be expected even when *VE*_*asym*_ is low or negative. A vaccine with a high *VE*_*asym*_ would work predominantly by preventing infection (high *VE*_*in*_, low *VE*_*pr*_), whereas a vaccine with a low *VE*_*asym*_ would work predominantly by preventing symptoms (low *VE*_*in*_, high *VE*_*pr*_).

Second, we estimated that the ChAdOx1 weekly PCR-measured *VE*_*in*_ was underestimated by 8.1% (Trial 50.9%, Model 59.0%) and *VE*_*asym*_ by 12.8% (Trial 14.6%, Model 27.4%). The *VE*_*pr*_ calculated from the trial reported *VE*_*in*_ and *VE*_*sym*_ would therefore be an overestimation (Calculation 43.6%, Model 31.5%). However, a wide range of values are compatible with the reported trial results when considering stochastic variation and parameter uncertainty. The small sample size informing the Ad26.COV2.S *VE*_*asym*_ estimate and parameter uncertainty for the test specificity, infection rate and adherence to PCR testing, in particular, reduced the precision of our uncertainty intervals. The true *VE*_*in*_ may range between 38.4% and 77.1%, and *VE*_*pr*_ between −20.7% and 55.0%. Given the strong bias that can be caused by reduced test specificity and a high frequency of testing, it would not be unreasonable for the true *VE*_*in*_ to be closer to our upper uncertainty interval, especially considering that effectiveness studies have estimated greater protection against infection than the trial [27, 28]. For Ad26.COV2.S, our model suggests that the true *VE*_*in*_ lies between 49.8% and 80.7%, with a best estimate of 70.9%. Although this indicates a negative *VE*_*pr*_, we believe this is unlikely and rather explained by the small sample size informing the trial *VE*_*asym*_ estimate.

We explain these overall expected differences by 4 likely biases acting in the COVID-19 trials.

1. A lower probability of detecting asymptomatic infections relative to symptomatic infections leads to overestimation of *VE*_*in*_ if the vaccine protects against symptom development. For these vaccines, some infections will be prevented from causing symptoms, so will be less likely to be detected. *VE*_*pr*_ would be mistaken for *VE*_*in*_, so *VE*_*in*_ would be overestimated. Both conditions for this bias are likely to be satisfied in the COVID-19 trials, as virological and serological testing approaches are less sensitive to asymptomatic infections [29, 30]. This bias is likely to have influenced the ChAdOx1 *VE*_*in*_ estimate, however we expect it was overridden by a competing downward bias. It is important to note that this bias does not affect *VE*_*asym*_ or *VE*_*sym*_.
2. Imperfect test sensitivity and specificity bias estimates toward zero, with greater bias with higher frequency of testing, lower infection rate and for VE based on cumulative incidence rather than incidence rates. This bias is caused by false positives in both trial arms and is greater with higher ratios of false positives to true positives [6]. It has potential to affect all VE outcomes but is likely to affect estimates of *VE*_*asym*_ and *VE*_*in*_ more than *VE*_*sym*_, because the combined probability of experiencing symptoms consistent with COVID-19 when not infected with SARS-CoV-2 and receiving a false positive test is low. Regression analysis showed that this was the predominant factor leading to underestimation of *VE*_*asym*_ and *VE*_*in*_ for both ChAdOx1 and Ad26.COV2.S in our model. As the bias is greater when testing is frequent, even a test with high specificity could bias the estimated ChAdOx1 *VE*_*in*_ and *VE*_*asym*_ noticeably toward zero. This could explain such contrasting trial reported *VE*_*asym*_ estimates between ChAdOx1 and Ad26.COV2.S, despite their similar platforms and neutralizing antibody responses [31, 32].
3. A build-up of natural immunity from undetected asymptomatic infections contributes a small downward bias in *VE*_*sym*_ for infection-blocking vaccines and a small upward bias for disease-blocking vaccines. For an infection-blocking vaccine, the proportion of infections that are asymptomatic is unaltered by the vaccine. Therefore, the rate at which immunity from asymptomatic infections accumulates, relative to the detection of symptomatic infections, is equivalent across trial arms, leading to an underestimation of *VE*_*sym*_ [8, 33]. Yet for disease-blocking vaccines, a greater proportion of infections in the vaccine arm will be asymptomatic, accelerating the acquisition of immunity from undetected infections and introducing a conflicting upward bias. Our model and real-world effectiveness studies suggest the COVID-19 vaccines protect against both infection and symptoms [27, 34]. Therefore we expect the overall direction of this bias to be toward zero, and for its magnitude to be greater for vaccines with higher *VE*_*in*._
4. Decreasing VE by age will bias estimated *VE*_*sym*_ downward and *VE*_*asym*_ upward, unless adjusted for age. This is due to older participants contributing more to *VE*_*sym*_ estimates than younger participants, who contribute more to *VE*_*asym*_ estimates. This bias is dependent on the probability of symptoms increasing with age, for which there is mixed evidence [35–37]. However, it should be considered when interpreting estimates based on different subgroups, such as *VE*_*asym*_ estimates based on a subgroup with serological data when *VE*_*sym*_ is based on the total population.

These biases also apply to effectiveness studies, based on cohort or case-control designs. Notably, the bias arising from differential detection of asymptomatic and symptomatic infections will likely be greater in real-world studies, where asymptomatic testing is less rigorous. This should be considered when comparing real-world and trial reported estimates, as it could lead to greater bias toward overestimation of *VE*_*in*_ in effectiveness studies.

Limitations to our analysis include uncertainties over parameter estimates. There is limited evidence on serology and PCR test sensitivities for asymptomatic infections, and how these change over time. As we show, differences in test sensitivity by symptom status can lead to overestimation of *VE*_*in*,_ so further studies are needed to clarify the potential role of this bias. We also did not consider the vaccines’ effects on viral load and how this alters virological and serological test sensitivity. Multiple COVID-19 vaccines reduce SARS-CoV-2 viral load [18, 38], and lower load infections are less likely to lead to seroconversion [39]. Therefore serology-based efficacy estimates may be more representative of high viral load infections than all infections, and may be comparable to estimates based on DNA sequenced swabs, which must exceed a threshold viral load to be sequenced. Finally, we do not consider point prevalence estimates from single time point PCR swabs, however this has been explored elsewhere [9, 10].

In conclusion, multiple biases have the potential to influence COVID-19 VE estimates, with their direction and magnitude dependent on the vaccine properties and testing strategies. These biases may explain differences between the ChAdOx1 and Ad26.COV2.S trial estimates despite similar vaccine platform technologies, and should be considered when interpreting efficacy and effectiveness study results as they are reported for these and other vaccines.

## Supplementary Data

Supplementary materials are available at *Clinical Infectious Diseases* online. Consisting of data provided by the authors to benefit the reader, the posted materials are not copyedited and are the sole responsibility of the authors, so questions or comments should be addressed to the corresponding author.

ciab914_suppl_Supplementary_Materials

## Notes

***Financial support.*** This work was supported by payments made to the Imperial College London, MRC Centre for Global Infectious Disease Analysis (grant number MR/R015600/1), jointly funded by the UK Medical Research Council (MRC) and the UK Foreign, Commonwealth & Development Office (FCDO), under the MRC/FCDO Concordat agreement and is also part of the EDCTP2 programme supported by the European Union. This work was also supported by the MRC Doctoral Training Partnership [to L. R. W.] and the Imperial College President’s Scholarship (to L. R. W.).

***Potential conflicts of interest.*** L. R. W. is currently a paid, part-time contractor for Gilead Sciences, working exclusively on human immunodeficiency virus (HIV) antiretroviral therapy post-licensure studies, outside of the submitted work and reports an unpaid position reviewing COVID-19 vaccine effectiveness studies for the World Health Organization COVID-19 Vaccine Effectiveness Working Group. C. A. D. reports payments made to Imperial College London, from UK National Institute for Health Research (NIHR) (Vaccine Efficacy Evaluation for Priority Emerging Diseases: PR-OD-1017-20007) and payments made to University of Oxford, UK NIHR (HPRU in Emerging and Zoonotic Infections: NIHR200907) during the submitted work; payments for book co-authored with Sir David Cox: *Principles of Applied Statistics* from Cambridge University Press and payments for book co-authored with Neil Ferguson: *Statistical Aspects of BSE and vCJD: Models for Epidemics* from Chapman & Hall/CRC Press; payments for consulting on the Defra-funded project: Developing a surveillance system to report tuberculosis in cattle herds exposed to badger control in England—SE3131 from UK Defra via UK Animal and Plant Health Agency (APHA); payment for honorarium for the 2020 Bradford Hill Lecture from London School of Hygiene and Tropical Medicine; unpaid trustee role as a member of the Council of the Royal Society, unpaid trustee role as the Vice President for External Affairs of the Royal Statistical Society (RSS), trustee role as a member of Governing Body (part of University of Oxford position) for St. Peter’s College, Oxford; unpaid position developing and evaluating possible vaccine effectiveness studies for World Health Organization R&D Blueprint, payment for review of grant proposals from Science Foundation Ireland, and payment for participation in the Dengue Expert Advisory Panel (DEAP) for Singapore National Environment Agency. N. C. G. reports being a member of the WHO SAGE COVID-19 vaccines working group. N. M. F. reports being PI of a grant funding the NIHR Health Protection Research Unit for Mathematical Modelling and Health Economics, co-PI of a grant funding COVID-19 modeling from UK Research and Innovation (UKRI), and PI of a philanthropic grant funding the Jameel Institute of Disease and Emergency Analytics at Imperial College during the submitted work; co-PI on a grant modeling antivirals against dengue from Janssen Pharmaceuticals, PI of a grant from BMGF funding the Vaccine Impact Modelling Consortium from Bill and Melinda Gates Foundation, PI of a grant from Gavi, funding the Vaccine Impact Modelling Consortium from Gavi, the Vaccine Alliance, outside of the submitted work; consultancy work for the World Bank Group on infectious disease threats that ceased in 2019 from the World Bank; payment for sitting on a grant panel and an advisory board for the Wellcome Trust (now ceased); travel expenses for WHO meetings from the World Health Organization; sat on an advisory board for Takeda in relation to their dengue vaccine and received no honorarium, gifts or expenses of any kind; and is senior editor for the journal *eLife*. All authors have submitted the ICMJE Form for Disclosure of Potential Conflicts of Interest. Conflicts that the editors consider relevant to the content of the manuscript have been disclosed.
